# A collection of intrinsic disorder characterizations from eukaryotic proteomes

**DOI:** 10.1038/sdata.2016.45

**Published:** 2016-06-21

**Authors:** Michael Vincent, Santiago Schnell

**Affiliations:** 1 Department of Molecular & Integrative Physiology, University of Michigan Medical School, Ann Arbor, Michigan 48109-0622, USA; 2 Department of Computational Medicine & Bioinformatics, University of Michigan Medical School, Michigan 48109-2218, USA; 3 Brehm Center for Diabetes Research, University of Michigan Medical School, Ann Arbor, Michigan 48105-1912, USA

**Keywords:** Proteome informatics, Protein databases, Proteome

## Abstract

Intrinsically disordered proteins and protein regions lack a stable three-dimensional structure under physiological conditions. Several proteomic investigations of intrinsic disorder have been performed to date and have found disorder to be prevalent in eukaryotic proteomes. Here we present descriptive statistics of intrinsic disorder features for ten model eukaryotic proteomes that have been calculated from computational disorder prediction algorithms. The data descriptor also provides consensus disorder annotations as well as additional physical parameters relevant to protein disorder, and further provides protein existence information for all proteins included in our analysis. The complete datasets can be downloaded freely, and it is envisaged that they will be updated periodically with new proteomes and protein disorder prediction algorithms. These datasets will be especially useful for assessing protein disorder, and conducting novel analyses that advance our understanding of intrinsic disorder and protein structure.

## Background & Summary

Despite the absence of a specific tertiary structure, intrinsically disordered protein regions are now understood to have biochemical functions, including serving as post-translational modification sites, effectors, entropic linkers between structured domains, in addition to many others^1^. Furthermore, multiple lines of evidence have shown that proteins and protein regions exhibiting intrinsic disorder play critical functional roles in a variety of cellular processes^2–11^. However, intrinsic disorder continues to pose significant experimental challenges^12^, and as a result, computational resources continue to serve as valuable tools for its investigation.

Large-scale studies at the proteomic level have provided a high volume of insightful information regarding the prevalence of disorder in multiple kingdoms of life^13–17^. In addition, numerous databases containing disorder residue annotations exist, with many aiding in the organization of annotations from multiple prediction algorithms and relevant experimental information^18–24^.

Although data collection and organization efforts have improved during the last decade, there remains a large amount of variability in the format, detail, quality, and availability of proteomic disorder datasets. In many cases it is also unclear whether algorithm-dependent sequence eligibility screening is performed, which is necessary when uncertainty exists regarding the identity of one or more residues in a protein sequence as the handling of this uncertainty varies greatly among disorder prediction algorithms. For example, some algorithms truncate unsupported residue types (such as B, J, O, U, X, and Z) during sequence processing, resulting in altered sequences and erroneous disorder annotations. Thus, inadequate eligibility screening could jeopardize the accuracy of proteomic disorder datasets that have a substantial number of partially defined sequences, such as the *Homo sapiens* UniProt reference proteome file which contains 7,082 of 68,485 (10.3%) sequences of this nature.

Here, we release a database containing IUPred and DisEMBL disorder annotations, as well as consensus annotations, calculated disorder parameters, and protein existence information for all completely defined protein sequences contained in ten eukaryote reference proteome files. The database can be used to standardize quantitative indicators of intrinsic disorder in a protein using descriptive statistics of disorder for the proteome in which it resides. In addition, the proteomic datasets within the database provide reliable, highly organized parameters and intrinsic disorder calculations that can be used for subsequent statistical analyses and investigations that further our understanding of both disorder and protein structure.

## Methods

### Summary

**Figure 1:**
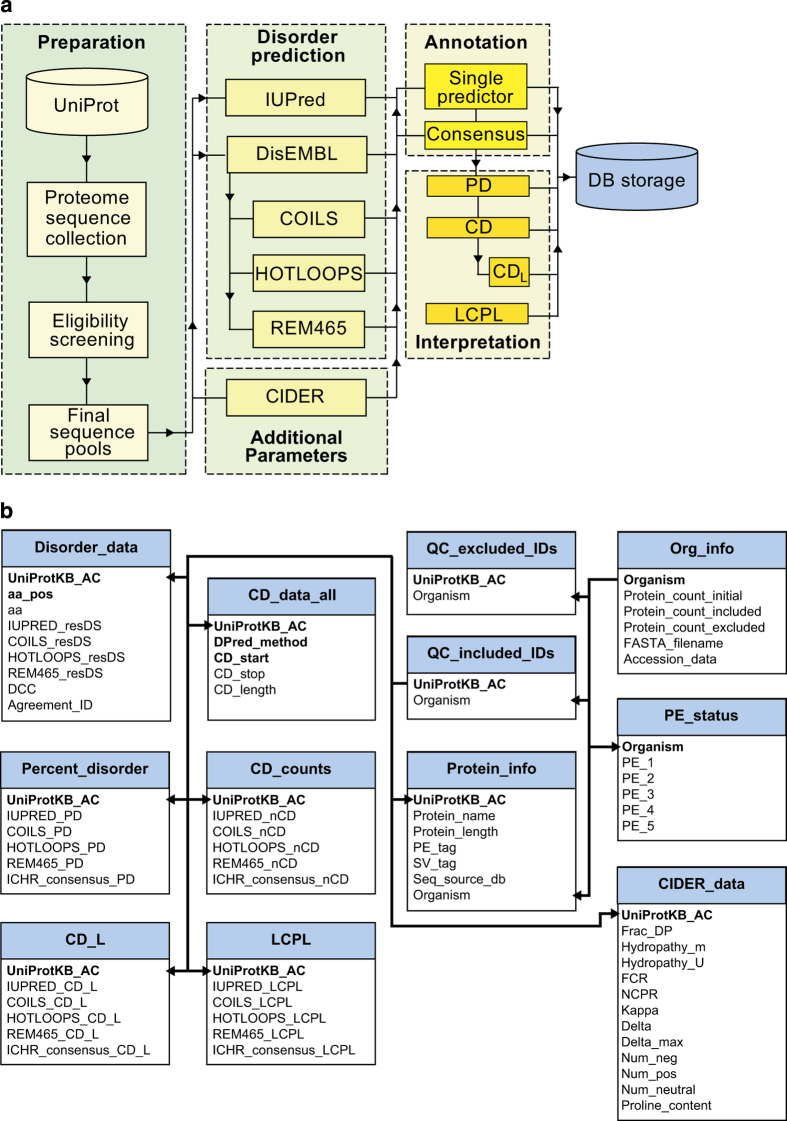
Workflow and database schema. (**a**) Data collection and processing procedures, consisting of protein sequence preparation, computational disorder prediction, residue disorder annotation, disorder feature interpretation and/or calculation, and storage in the final database. (**b**) Schema of the final database. The primary key of each table is displayed in bold. Multiple bolded items represent a composite primary key.

In this section we have described our data collection and processing procedures for obtaining residue-by-residue disorder annotations, disorder descriptive statistics, and other physical parameters relevant to protein disorder (such as hydropathy, and charge mixing). Furthermore, this information also includes consensus disorder annotations and protein existence information. A diagram of the data collection and processing workflow has been displayed in Fig. 1a.

### Protein sequence collection and quality screening

Protein sequences for the following ten common model eukaryotic proteomes were collected from UniProt: *Arabidopsis thaliana, Caenorhabditis elegans, Chlamydomonas reinhardtii, Danio rerio, Dictyostelium discoideum, Drosophila melanogaster, Homo sapiens, Mus musculus, Saccharomyces cerevisiae,* and *Zea mays*^25^. The UniProt proteome identifier and accession date for each proteome is displayed in Table 1. Many disorder prediction algorithms have not been designed to predict disorder in sequences containing undefined residues. Due to the variability in handling of undetermined/unknown, ambiguous, and/or unique amino acids (B, J, O, U, X, Z) by disorder prediction algorithms, proteins containing these residues were excluded from our analysis. A complete list of UniProt accession numbers that have been deemed eligible and ineligible for each organism has been provided in the published database.

### Disorder prediction

Two reputable disorder prediction algorithms, IUPred-L (version 1.0)^26,27^ and DisEMBL (version 1.4)^28^, were used to assess disorder in each of the ten eukaryotic proteomes included in our investigation. Aside from the positive reputation of these algorithms, IUPred and DisEMBL were also chosen due to their physicochemical premise and the fact that they do not rely on sequence alignment to predict disorder. Briefly, IUPred predicts disorder from an energetics standpoint and assesses the capacity of a protein to form interresidue contacts that would serve to provide structural stability^26,27^. On the other hand, DisEMBL provides three methods based on neural networks for disorder prediction: Coils (DisEMBL-C), Hotloops (DisEMBL-H), and REM465 (DisEMBL-R). DisEMBL-C predicts residues belonging to coils/loops as disordered, which excludes residues belonging to α-helix, 3_10_-helix, or β-strand secondary structures, whereas DisEMBL-H predicts disorder by assessing the subset of the DisEMBL-C-predicted population that have high α-carbon temperature factors^28^. DisEMBL-R is a neural network that has been trained using missing X-ray crystallography coordinates from Protein Data Bank files, which assumes disorder accounts for the missing residue information^28^. For additional details regarding IUPred and DisEMBL, please refer to the original publications in which they were released^26–28^. For each algorithm, residues were marked as either ordered or disordered by comparing disorder scores to the published default threshold values for IUPred^26,27^ and DisEMBL^28^. In addition to single predictor annotations, consensus annotations have been provided as well. Residues were classified as disordered or ordered by ‘consensus’ if all individual prediction algorithms were in agreement regarding the disorder classification. Lastly, disorder content was calculated as the percentage of disordered residues contained within a protein.

### Continuous disorder protein populations and metrics

The *theoretical* minimum length of a region exhibiting continuous disorder (CD) is two amino acids. Given the absence of an objectively determined minimum length, we have chosen to use the aforementioned theoretical minimum to define CD regions in our dataset. In many cases, a protein contains multiple CD regions of varying lengths. Our database includes information regarding the boundaries of each CD region predicted to exist in a protein by each individual disorder prediction algorithm, and also includes information regarding CD regions for which there is consensus agreement. Furthermore, it is often helpful to assess the longest CD region (CD_L_) of a protein, as well as the percentage of the primary sequence length of a protein that is accounted for by the CD_L_. The latter metric is referred to as the LCPL^17^, and it can serve as a more reliable indicator of a significantly long CD region in proteins with exceptionally long primary sequences. Thus, our database not only includes information regarding each CD region contained in a protein, but we have also recorded the CD_L_ and the LCPL for each CD-exhibiting protein in order to provide alternative metrics for gauging the significance of a CD region. Threshold protein lengths for gauging when to use the LCPL instead of the CD_L_ can be found in Vincent *et al.*, 2016 (ref. 17). Importantly, note that if none of the disorder prediction algorithms predict CD in a given protein, then that protein will not appear in any of the CD-based tables. However, if one or more, but not all, prediction algorithms predict continuous disorder for a given protein, ‘N/A’ will be displayed for the algorithms doubting the existence of CD in the protein.

### Collection of additional physical parameters relevant to protein disorder

To compliment the aforementioned disorder predictions, we have also provided additional parameters relevant to protein disorder. Briefly, these parameters characterize the fraction of disorder promoting amino acids, the hydropathy, and charge distribution for a given sequence, and have been calculated using localCIDER version 0.1.7 (Classification of Intrinsically Disordered Ensemble Regions). The localCIDER software was obtained from http://pappulab.github.io/localCIDER/. A brief description of the CIDER-calculated parameters included in our database can be found below in the Data Records section.

### Code availability

Local copies of the IUPred and DisEMBL prediction algorithms are available for download at http://iupred.enzim.hu/Downloads.php and http://dis.embl.de/html/download.html, respectively. Please refer to the IUPred and DisEMBL websites for policies governing their use. Internal software used to collect, process, and analyse the data was written in Python 2.7.10 and is available upon request.

## Data Records

### Summary

The data resulting from the collection and processing procedures described above in the methods section have been made available as a SQLite3 file (Data Citation 1). The database consists of a total of 12 tables, which provide both general descriptive metrics regarding the proteomic protein populations under study as well as more detailed information including residue-by-residue disorder annotations, calculated features (disorder content, CD_L_, and LCPL), and other relevant physical parameters (such as hydropathy, and charge distribution.). Please refer to Figure 1b for a representation of the database schema.

### Organism data

The database includes the following information for each of the ten eukaryotes in which disorder was examined: the specific UniProt reference proteome FASTA file name, the date that the FASTA file was accessed, the number of included protein sequences, and the number of excluded protein sequences. The UniProtKB accession ID has also been provided for all included and excluded proteins. Protein inclusion and exclusion criteria have been discussed in detail in Methods.

### Residue-by-residue disorder score annotation data

**Figure 2:**
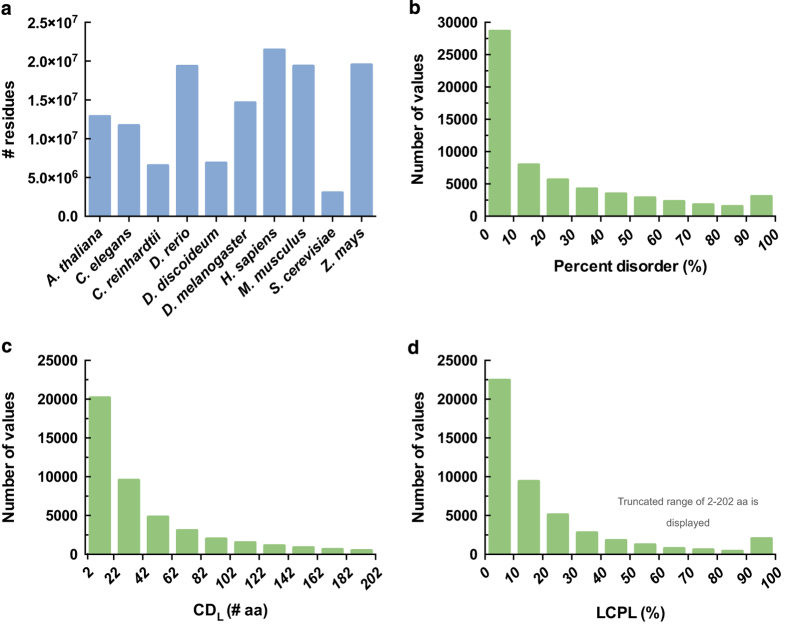
Annotation counts and example disorder feature distributions. (**a**) The number of annotated residues for each proteome. (**b**–**d**) Histograms displaying example distributions of percent disorder (**b**) the longest continuous disordered region (CD_L_) (**c**) and the longest continuous disordered region percentage of length (LCPL) (**d**). Only IUPred predictions for the *Homo sapiens* proteome are shown in this figure. Note that the continuous disorder (CD) distributions are only for the subset of the proteome exhibiting CD, as the minimum possible length for a CD region is two amino acids and therefore proteins lacking CD must be excluded from the CD analysis. Furthermore, although the CD_L_ in *Homo sapiens* ranges from 2-4,638 amino acids, a truncated range of 2-202 amino acids is displayed for the CD_L_ distribution presented in (**c**) in order to facilitate data visualization, as approximately 95% of the data falls within this range.

For each sequence in each of the ten proteomes selected for analysis, detailed records of the disorder score output from each prediction algorithm have been reported for each residue in the ‘Disorder_data’ table. The position in the sequence and residue identity has been indicated as well. Binary disorder and order classifications can be obtained by comparing the algorithm-specific disorder scores against the corresponding default threshold values published in the literature^26–28^. Nevertheless, between the ten eukaryotes our database contains annotations for 135,302,222 residues (Fig. 2a).

In addition to single-predictor disorder score annotations, a disorder classification count (DCC) and agreement ID has been reported for each residue as well. The DCC simply represents the number of component prediction algorithms classifying the residue as disordered. The DCC ranges from zero to four, with four representing the total number of disorder prediction methods and therefore represents the maximum number of methods that can classify a residue as disordered in this study (for example, a DCC of three indicates that three of the four prediction methods are in agreement). On the other hand, the agreement ID is simply a string between one and four characters in length indicating the identity of the disorder prediction methods in agreement regarding the residue-level classification of disorder. Agreement regarding a disorder classification exists if a minimum of two of the four disorder prediction methods classifies the residue as disordered. For example, an agreement ID of ‘ICHR’ indicates that all four methods, IUPred (I), DisEMBL—C (C), DisEMBL—H (H), and DisEMBL—R (R) agree that the residue is disordered. However, if only a single method classifies a residue as disordered, an agreement ID of ‘NA’ has been assigned to the residue to indicate ‘no agreement’. Lastly, if zero of the four disorder prediction methods have classified the residue as disordered (i.e., all four methods agree that the residue is ordered), an ‘O’ agreement ID has been assigned to the residue indicating consensus order (it should also be noted that an ‘O’ agreement designation corresponds to a DCC of zero, as the DCC focuses on multi-predictor agreement regarding the classification of disorder).

### Descriptive disorder feature data

While disorder scores represent the raw output from the IUPred and DisEMBL disorder prediction algorithms, proteomic investigations of disorder typically analyse distributions of descriptive disorder features that are derived from disorder score interpretations. Additionally, these interpreted disorder features are also very useful for analysing disorder in individual proteins. Thus, our database also includes information regarding the percentage of disordered residues and CD regions^17^. Regarding the latter, detailed information has been included that records all CD regions (including residue position boundaries and length) and the number of CD regions found in a protein, as well as the CD_L_ and LCPL. The distribution of IUPred-determined percent disorder, CD_L_, and LCPL has been displayed for the *Homo sapiens* proteome as an example (Fig. 2b–d).

### Additional physical parameters relevant to protein disorder

Aside from disorder annotations and their interpreted descriptive statistics, our database also provides insightful parameters to further characterize protein disorder. Specifically, for each protein we have included the fraction of disorder promoting residues^29^, the mean hydropathy^30^, the Uversky hydropathy^30,31^, the fraction of charged residues (FCR), the net charge per residue (NCPR), the kappa (κ) parameter describing the extent of amino acid charge segregation in a sequence^32^, the δ and δ_max_ parameters used to calculate κ^32^, and the proline content (note that as stated by the official CIDER documentation, κ may be inaccurate for sequences with a proline content >15%). The κ parameter ranges between zero and one, with κ approaching one indicating a greater degree of segregation of positive and negative charges in the sequence, whereas κ values closer to zero indicate a greater degree of mixing between positive and negative charges^32^. Furthermore, the number of negatively charged, positively charged, and neutral amino acids have been reported for each sequence as well. For details regarding each of these parameters, please refer to the official CIDER documentation located at http://pappulab.wustl.edu/CIDER/.

### Limitations and potential for expansion

As previously stated, the objective of this database is to provide a reliable collection of disorder annotations, statistics, and relevant disorder parameters from protein amino acid sequences in common eukaryotic proteomes. While we believe this information is highly valuable, we acknowledge that it has limitations and room for expanding the information available in our dataset. Users are encouraged to combine the disorder annotations and parameters provided here with external resources relevant to their specific research investigations. Our database can be easily combined with external data sets, such as those providing structural and/or post-translational modification annotations, in order to facilitate a variety of computational and experimental projects.

Two limitations of the database include the number of supported disorder prediction algorithms and the format of the CIDER-based parameters. Regarding the former, we limited our protein disorder predictions to algorithms using physicochemical principles. While these algorithms predict disorder using various disorder definitions, disorder predictions might be improved through the addition of a larger number of algorithms, including algorithms predicting disorder using protein sequence alignment. As for the limitations pertaining to the CIDER parameters, these parameters cannot be readily used to classify intrinsically disordered protein regions in the format we provide. This is due to the fact that we have provided these parameters as per sequence annotations, rather than per residue annotations. Implementing algorithms with sliding window estimates from specific regions of the amino acid sequence would be useful for the classification of disordered regions. However, calculations of this nature cannot be practically included in a static database of this nature.

## Technical Validation

### Summary

Data regarding the performance and accuracy of IUPred and DisEMBL can be found in the original publications in which these disorder prediction algorithms were presented^26–28^. Algorithm considerations aside, the integrity of the datasets published in this paper largely depends on the quality of the protein sequences included. The present data descriptor only includes proteins eligible for assessment using IUPred and DisEMBL (our eligibility screening procedure is described below). In addition, UniProt protein existence information has been provided for all protein populations as well.

### Protein eligibility screening

**Figure 3:**
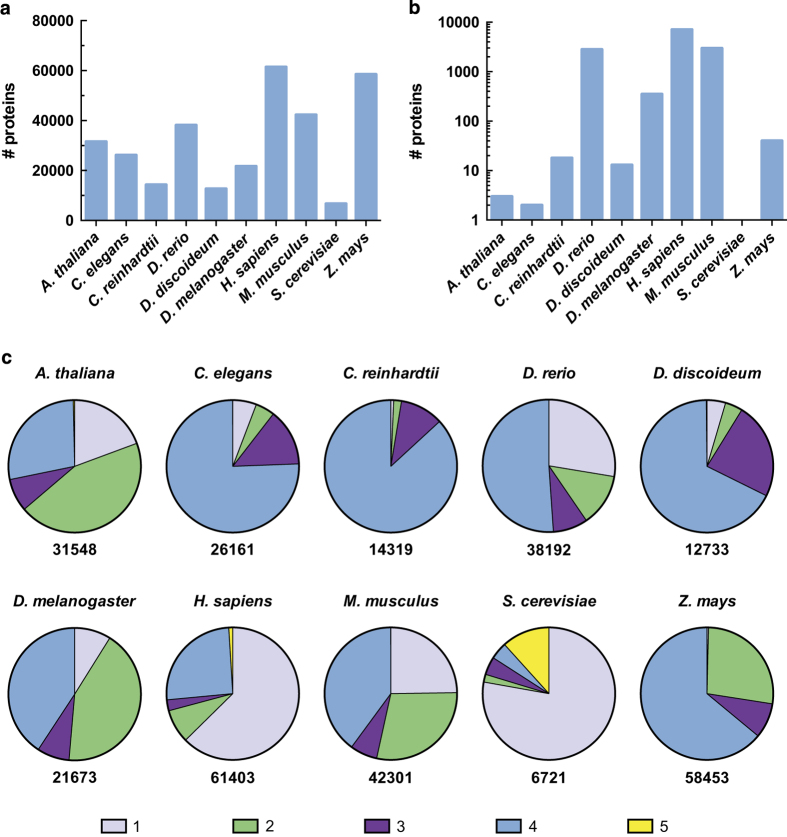
Sequence eligibility screening and protein existence information. The number of proteins from each proteome that has been included (**a**) and excluded (**b**) following eligibility screening is displayed (a log scale has been used in (**b**) to facilitate visualization of the data as fewer than 100 sequences were excluded for six of the ten proteomes). Within the included protein populations, the fraction of the population belonging to each of the five UniProt protein existence (PE) qualifiers is presented in (**c**). PE 1 and PE 2 indicate experimental evidence at the protein level and transcript level, respectively^25^. PE 3 indicates that the protein has been inferred from homology^25^. PE 4 indicates that the protein has been predicted, but evidence required for PE 1-3 classification is absent^25^. PE 5 indicates uncertainty regarding the existence of the protein^25^. The total number of eligible proteins for each proteome is displayed below each chart.

To ensure that only sequences deemed suitable for analysis with the aforementioned disorder prediction algorithms are included, an eligibility screening procedure was conducted prior to data collection in which proteins containing unsupported residue types were excluded from the input protein set. Importantly, if a protein was deemed ineligible for analysis by one disorder prediction algorithm it was excluded from the study population altogether (i.e., for inclusion in our study, a protein sequence must be eligible for analysis by both IUPred and DisEMBL). As described in Methods, proteins containing undetermined/unknown, ambiguous, and/or unique amino acids (B, J, O, U, X, Z) were excluded from our analysis. The total number of included and excluded proteins for each proteome has been displayed in Fig. 3a,b. Please note that our database includes sequences from both Swiss-Prot and TrEMBL entries, and the latter may include additional predicted isoforms that could increase redundancy^25^.

### Protein existence information

Information regarding the existence of the proteins has also been included in the released database and can aid in assessing the validity of the included protein sequences. Protein existence (PE) information provided in the header of UniProt reference proteome files has been recorded in the ‘PE_status’ table for all proteins included in our database. Briefly, UniProt defines the PE qualifiers of one, two, and three to indicate ‘experimental evidence at the protein level’, ‘experimental evidence at the transcript level’, and that a protein has been ‘inferred from homology’, respectively^25^. Additionally, a PE four qualifier describes a sequence that lacks evidence at either of the three aforementioned levels, whereas a PE five qualifier indicates uncertainty regarding the existence of the protein^25^. Please refer to the official UniProt documentation for additional details regarding the procedure used for assigning protein existence qualifiers. The large majority of the eligible sequences in the ten proteomes where found to be of PE qualifiers one through four, suggesting minimal uncertainty regarding the existence of the sequences comprising our datasets (Fig. 3c). Furthermore, only *Saccharomyces cerevisiae* was found to contain a substantial fraction of proteins with a PE five qualifier, which represents 11.7% of the entire population (Fig. 3c).

## Usage Notes

For completeness, the published database contains protein sequences from both UniProtKB/Swiss-Prot and UniProtKB/TrEMBL. Proteins with UniProt PE qualifiers of one through five have also been included in the database. Thus, users must take the appropriate measures to reduce the sequence redundancy and existence uncertainty within the published database. To decrease sequence redundancy, we encourage users to utilize information regarding the protein sequence source (UniProtKB/Swiss-Prot and UniProtKB/TrEMBL) and the protein existence (PE 1–5) that is contained within the database, along with external information from the UniProt Reference Clusters (UniRef).

This Data Descriptor inroduces a dataset with diverse potential applications, which include, but are not limited to, (1) theoretical quantitative studies seeking to find correlations between properties giving rise to intrinsic disorder, (2) structural assessments seeking to determine whether a protein of interest contains a significant long disordered region that may hinder crystallization, and (3) population statistics-based approaches aiming to assess whether the predicted disorder properties in a protein of interest are significant with respect to the rest of the proteome.

## Additional Information

**How to cite**: Vincent, M. & Schnell, S. A collection of intrinsic disorder characterizations from eukaryotic proteomes. *Sci. Data* 3:160045 doi: 10.1038/sdata.2016.45 (2016).

## Supplementary Material



## Figures and Tables

**Table 1:** Proteome sequence file information.

**Proteome**	**Proteome identifier**	**Accession date**
*Arabidopsis thaliana*	UP000006548	5/7/2015
*Caenorhabditis elegans*	UP000001940	5/7/2015
*Chlamydomonas reinhardtii*	UP000006906	7/6/2015
*Danio rerio*	UP000000437	7/6/2015
*Dictyostelium discoideum*	UP000002195	6/22/2015
*Drosophila melanogaster*	UP000000803	5/7/2015
*Homo sapiens*	UP000005640	5/7/2015
*Mus musculus*	UP000000589	5/7/2015
*Saccharomyces cerevisiae*	UP000002311	5/7/2015
*Zea mays*	UP000007305	7/6/2015
